# Hydrodynamics of Butterfly-Mode Flapping Propulsion of Dolphin Pectoral Fins with Elliptical Trajectories

**DOI:** 10.3390/biomimetics8070522

**Published:** 2023-11-03

**Authors:** Dan Xia, Zhihan Li, Ming Lei, Yunde Shi, Xiang Luo

**Affiliations:** School of Mechanical Engineering, Southeast University, Nanjing 211189, China; lzh809963603@163.com (Z.L.);

**Keywords:** hydrodynamics, butterfly mode, elliptical track, pectoral fins, self-propelled dolphin, numerical simulation

## Abstract

This article aims to numerically study the hydrodynamic performance of the bionic dolphin equipped with a pair of rigid pectoral fins. We use dynamic-grid technology and user-defined functions to simulate a novel butterfly-mode flapping propulsion of the fins. This pattern of propulsion is composed of three angular degrees of freedom including the pitch angle *ϕ_p_*, the azimuth angle *ϕ_a_* and the roll angle *ϕ_r_*, which can be divided into four stages for analysis within a single cycle. The stroke of one single pectoral fin can be approximated as an ellipse trajectory, where the amplitudes of *ϕ_a_* and *ϕ_p_*, respectively, determine the major and minor axes of the ellipse. The fluid dynamics involved in the specific butterfly pattern is mathematically formulated, and numerical simulation is conducted to investigate the propulsion quantitatively. The results show that the dolphin with a higher water striking frequency *f* can acquire higher propulsion speed and efficiency. Furthermore, the shape of the ellipse trajectory under different conditions could also have different propulsion effects. The periodic generation and disappearance of vortex structures in the butterfly flapping mode show the evolution process of fluid flow around a pair of pectoral fins, which reveals the influence of motion parameters on fluid dynamics under different working conditions.

## 1. Introduction

In recent years, human’s emphasis on the field of bionics has promoted the innovative development of fish-like underwater vehicles [1,2,3,4]. Their mobility plays a decisive role in oil and gas exploration, scientific deep-sea surveys and mapping of complex seabed. As a basic component of the propulsion system, the caudal fin has always been the main focus and has been widely studied in both bionic experiments [5,6,7,8] and numerical simulations previously [9,10,11,12]. However, compared to the caudal fins, the pectoral fins play a more important role in posture adjustment. Can a novel motion mode be designed based on the traditional fin flapping law? This research is inspired by human butterfly swimming and the flexible use of pectoral fins on both sides of the dolphin. In the previous studies of self-propulsion, pectoral fins mostly assisted the propulsion of caudal fins, so the hydrodynamics of the butterfly-mode flapping propulsion with only the pectoral fins has not been systematically studied and understood.

Representative examples of aquatic creatures that rely primarily on pectoral fins for propulsion include batfish and cownose rays. Unlike the conventional fishes with the body and/or caudal fin (BCF), these creatures possess a structure characterized by the body and symmetrical pectoral fins. This unique anatomical configuration enables them to thrive in underwater environments and perform a diverse range of swimming maneuvers. This distinct mode of movement is commonly referred to as median and/or paired fin (MPF) propulsion. Many biomimetic studies based on the motions of pectoral fins have been carried out in a systematic manner. Kato and Liu [13] installed mechanical pectoral fins on both sides of the developed underwater vehicle and systematically studied the hydrodynamic performance through experiments. Behbahani [14] proposed a flexible passive joint for connecting a robotic fish body and a pair of pectoral fins, which maximized the efficiency during the recovery stroke. Singh [15] compared the results of the pre-motion parameters of the paddling robot from both theoretical and experimental perspectives. The study found errors between the theoretical and actual values, which were caused by fin tip loss or induction speed. Santo [16] studied the oscillations of the extended pectoral fins of batfish in the test tank by varying the beat speed and frequency. Arastehfar [17] evaluated the influence of the sweep angle on the propulsion based on a fish-like robot with batwing-shaped pectoral fins.

Recently, there have been some numerical studies of underwater propulsion based on pectoral fins. Chen [18] numerically studied the hydrodynamic law of the swing amplitude of pectoral fins for self-propulsion based on the batoid model. Yu [19] explored the relationship between pectoral fin vortices and body traveling wave propulsion, and found the symmetrical pectoral fin movement is beneficial to reduce the lateral energy loss and improve the propulsion performance. Li [20] focused on the hydrodynamic characteristics of pectoral fins of different flexibilities, and obtained the best combination of parameters for high propulsion performance. Sampath [21] designed a tandem flapping pectoral fin and numerically changed the stroke phase offset of the front and rear fins to study the behavior of fluid. Weng [22] applied a variety of rays and membranes to create a deformable bionic pectoral fin and demonstrated the hydrodynamic performance. Shoele [23] used several skeleton-enhanced fin rays to form pectoral fins and numerically simulated the propulsion using two degrees-of-freedom (DoF) motions. Xu [24] used overset grids to discretize the bionic fish equipped with rigid pectoral fins and realized self-propulsion and self-steering based on the flapping motion of pectoral fins. From the above simulation results, the application of pectoral fins can greatly improve the maneuverability of underwater vehicles. However, these studies mostly imitate the existing flapping patterns of pectoral fins found in nature. As a result, a novel butterfly flapping mode of rigid pectoral fins is proposed in this study to further improve self-propulsion.

In this work, a bionic dolphin is selected as a swimming carrier, and the butterfly flapping motions of its pectoral fins are designed to form an elliptical trajectory. To verify the efficiency, stability and innovativeness of the rigid pectoral fins in self-propulsion, the hydrodynamic characteristics of the butterfly flapping motion are studied. Necessary numerical simulation is conducted to help pave the way for the design of the robotic dolphin. While keeping the body of the dolphin undeformed, the designed joint angular trajectories are used to quantitatively control the associated joint motions of rigid pectoral fins. Then the self-propulsion performance of the bionic dolphin in 3D space is evaluated based on numerical simulation. Finally, the hydrodynamic parameters and the 3D vortex structure are analyzed in detail, which helps explain the influence of the pectoral fins’ regular paddling motions on the performance of propulsion.

## 2. Materials and Methods

### 2.1. Computational Models and Grids

**Figure 1:**
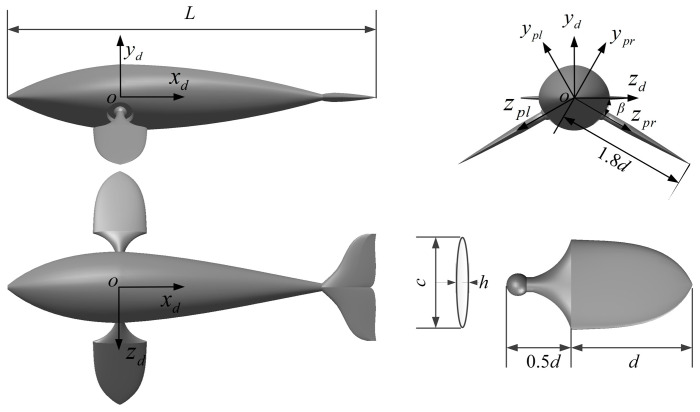
Physical model of the dolphin with its pectoral fins on both sides.

The computational model of the bionic dolphin equipped with a pair of rigid pectoral fins is shown in Figure 1. The pectoral fins are attached to the body of dolphin through spherical joints on both sides. To describe the butterfly flapping motion of the pectoral fins, we define the symmetrical coordinate system *ox_pl_y_pl_z_pl_* and *ox_pr_y_pr_z_pr_* for the orientation of the left and right pectoral fins, respectively, and *ox_d_y_d_z_d_* for the body of dolphin. If we take the right pectoral fin as an example, the design of pectoral fin is symmetrical about the *y_pr_z_pr_* plane and *x_pr_z_pr_* plane. In Figure 1, *c* is the span length of the pectoral fin, d is the chord length, the length of the handle is 0.5 *b* and the cross section is an ellipse with thickness *h*. In terms of the overall dimension, the length of the dolphin is *L*, the length from the center of mass of the dolphin to the tip of the pectoral fin is 1.8 *b*, and the angle between *oz_pr_* and *oz_d_* is *β* = 30° at the beginning.

**Figure 2:**
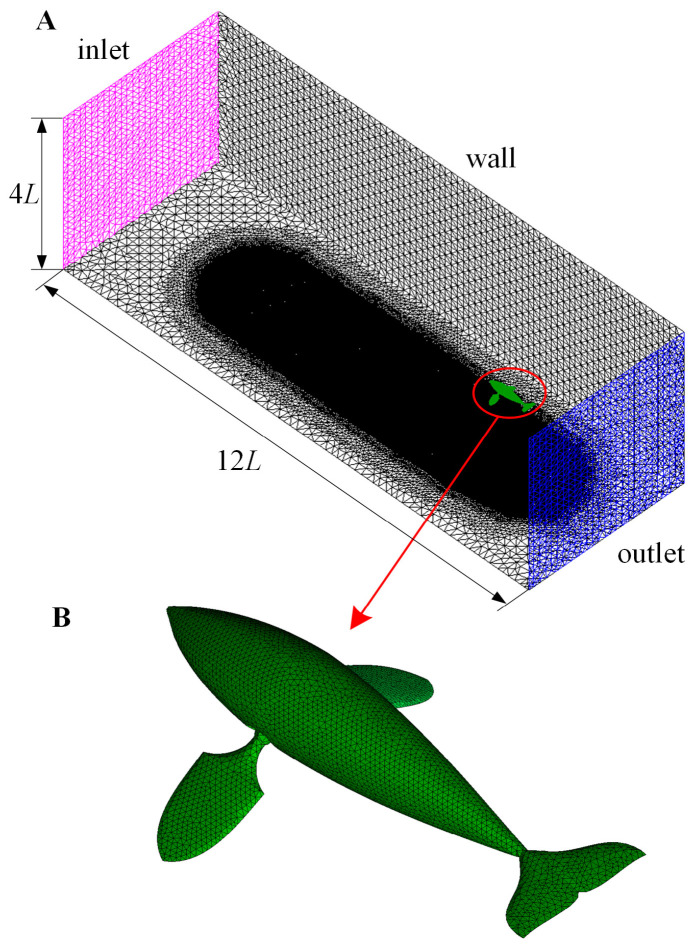
Grids for the water tank and the dolphin. (**A**) Size and boundary settings of the water tank; (**B**) Meshing method for the dolphin surface.

The physical size of the swimming domain with the related grid division are shown in Figure 2. The overall size of the water tank is 12 *L* × 4 *L* × 4 *L*, providing enough space for the dolphin to achieve self-propulsion. The bionic dolphin is placed at the center of the *y_b_z_b_* plane, and the dolphin’s tail is 1 *L* away from the outlet plane along the *ox_b_* axis direction. The paddling of pectoral fins is a highly stretchable motion process, so we use an easy-to-adjust tetrahedral grid to disperse the entire domain, in which the density of the grid around the dolphin is gradually refined to capture the detailed motion of the dolphin and structure of flow field. In Figure 2B, the surface of the dolphin is discretized into a uniform triangular grid. In a large number of previous studies [25,26], we used this meshing method to simulate the flexible swing of various fins to achieve underwater swimming. For the boundary conditions of inflow and outflow, we set zero velocity and pressure gradient for both the inlet and outlet boundary.

### 2.2. Kinematics of Pectoral Fins

**Figure 3:**
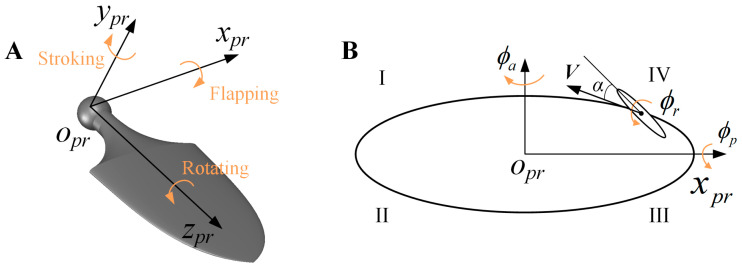
Movement description of the right pectoral fin in one cycle (**A**) The 3-DoFs angle decomposition; (**B**) The elliptic trajectory.

The pectoral fins are the main driving module of the bionic dolphin undergoing the butterfly-mode flapping behavior. The right pectoral fin is chosen as the reference object shown in Figure 3. The basic kinematics of butterfly-mode flapping motion could be described as a composite movement, including flapping back and forth (azimuth), flapping up and down (pitch)and self-rotation (roll). The elliptic trajectory is divided into four sections (I, II, III and IV) for hydrodynamic analysis in the following paper.

In the body-attached coordinate system of the right pectoral fin, point *o_pr_* represents the starting pivot of the butterfly flapping mode, the *o_pr_x_pr_* axis is along the span direction of the pectoral fin (i.e., the longitudinal direction of the dolphin body), the *o_pr_y_pr_* axis is along the normal direction and the *o_pr_z_pr_* axis is along the chord direction of the pectoral fin. The kinematic model of the composite motion, including the pitch angle *ϕ_p_*, the azimuth angle *ϕ_a_* and the roll angle *ϕ_r_*, is defined as follows:

(1) $$\phi_{r}=\pi t/T$$

(2) $$\phi_{p}=\phi_{p\mathrm{int}}+\phi_{p\max}\cos(2\pi ft+\Delta \phi_{p})$$

(3) $$\phi_{a}=\phi_{a\mathrm{int}}-\phi_{a\max}\cos(2\pi ft+\Delta \phi_{a})$$

where *T* is the period of waves, *f* is the water striking frequency, *ϕ_p_*_max_ and *ϕ_a_*_max_ denote the amplitude of the pitch angle *ϕ_p_* and the azimuth angle *ϕ_a_*. *ϕ_p_*_int_, *ϕ_a_*_int_ and *ϕ_r_*_int_ represent the initial values. Δ*ϕ_p_* and Δ*ϕ_a_* represents the phase of the pitch and azimuth angle with respect to the roll angle. The trajectory of the butterfly flapping over a full cycle can be described as an ellipse, as shown in Figure 3B. The periodic change in the roll angle is an effective tool for varying the water attacking angle, and the combined movement of the pitch angle and the azimuth angle forms the trajectory of the ellipse shape. The formula for the elliptical trajectory is given as follows:

(4) $${\left(\frac{\phi_{a}-\phi_{a\mathrm{int}}}{\phi_{a\max}}\right)}^{2}+{\left(\frac{\phi_{p}-\phi_{p\mathrm{int}}}{\phi_{p\max}}\right)}^{2}=1$$

In the grid deforming region, the relative positions of the pectoral fins and the dolphin body are transferred iteratively over time, so that motion information can be exchanged between the different grids at each time step. To express the fin movement over time, the butterfly-mode flapping movement of the pectoral fins sequentially undergoes azimuthal, pitching and rolling movements over a time step, so all grid points are transformed in sequence through the following relationship.

**Figure 4:**
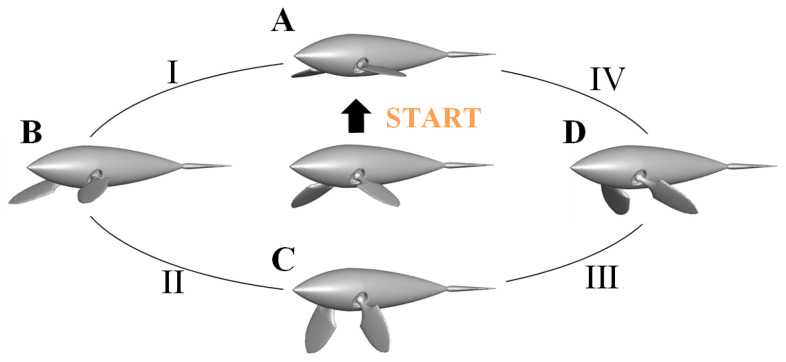
The prescribed kinematics of the butterfly motion within one cycle.

(5) [xaijknyaijknzaijkn]=[cos(ϕan)0sin(ϕan)010−sin(ϕan)0cos(ϕan)][xijkn-1−xpyijkn-1zijkn-1−zp]

(6) [xpijknypijknzpijkn]=[1000cos(ϕpn)sin(ϕpn)0−sin(ϕpn)cos(ϕpn)][xaijknyaijkn−ypzaijkn−zp]

(7) [xrijknyrijknzrijkn]=[cos(ϕrn)sin(ϕrn)0−sin(ϕrn)cos(ϕrn)0001][xpijkn−xpypijkn−ypzpijkn]

where *n* − 1 and *n* represent the (*n* − 1)-th and *n*-th time steps, respectively, the subscripts *i*, *j* and *k,* respectively, represent different mesh points. After a cycle of iterative calculations, we can obtain the complete water striking law based on the prescribed kinematics of butterfly motion shown in Figure 4.

Figure 4 illustrates the dolphin with the pectoral fins’ composite swing which experiences four distinct states (A, B, C and D) in one cycle of the elliptical track. These states correspond to *t* = (*n +* 0)*T*, (*n +* 1/4)*T*, (*n +* 2/4)T and (*n +* 3/4)*T*, where *n* is the number of swing cycles. I, II, III and IV, respectively, represent four transitional states to facilitate subsequent time history analysis.

### 2.3. The Governing Equations

We consider a 3D flow over the dolphin undergoing the self-propulsion. The governing equations of the fluid include the mass conservation equation and the Navier–Stokes momentum conservation equation written as

(8) ∇⋅u=0

(9) $$\rho \frac{\partial u}{\partial t}+\rho (u\cdot \nabla )u=-\nabla p+\mu \nabla^{2}u$$

where ***u*** are the fluid velocity, *ρ* is the density, *p* is the pressure, *μ* is the dynamic viscosity and ▽ is the gradient operator. To solve the equations in the domain containing the virtual dolphin, a no-slip boundary condition is needed to be imposed on the moving interface where the fluid velocity is equal to the dolphin velocity.

Due to the intricate shape of the dolphin and the rapid changes in curvature gradient, the flow around the dolphin exhibits a highly complex 3D pattern. When the bionic dolphin undergoes autonomous motion from rest to cruise, the Reynolds number (Re) fluctuates within an inertial range of 0~10^4^. Due to the difficulty in identifying the properties of the fluid, we adopt a transitional method in this research. In addition, Newton’s law of motion is used to describe the butterfly-mode propulsion of the swimmer:

(10) $$m\ddot{x}=F$$

where ***F*** is the fluid force vector, *m* is the dolphin mass which is depicted as the body volume multiplied by the water density, and $\ddot{x}$ is the swimming acceleration vector which is time-dependent due to the fluid force. The fluid force *F* are also expressed as follows:

(11) $$F=\int_{S}\overset{=}{\sigma }\cdot n_{s}dS$$

where ***σ*** is the normal stress tensor, ***n****_s_* is the unit vector along the normal direction and *dS* is the differential unit area along the dolphin surface.

### 2.4. Numerical Method and Validation Test

Numerical simulation has been widely used in our previous studies, including fishlike robots under self-propulsion, which indirectly verified the effectiveness of the 3-DoFs fluid–structure interaction (FSI) method. The solver ANSYS Fluent acts as the tool for computational fluid dynamics in the simulation process. We use the finite volume method to discretize the Navier–Stokes equations: The Green–Gauss cell-based method is applied for the setup of gradient interpolation in the spatial discretization, the second-order upwind scheme is adopted for the convective term, the first-order implicit Euler scheme is selected for the time discretization and a second-order central differencing scheme is employed for the diffusion term. We employ the second-order implicit scheme for the transient formation. The SIMPLE algorithm is used to calculate the pressure–velocity coupling of the continuity equation. Based on Newton’s motion equation, the user-defined function and dynamic mesh technology are applied for the butterfly-mode flapping movement, while the embedded DEFINE_CG_MOTION macro is hooked to the main code of the solver. During each step, mesh methods, including smoothing and remeshing, are applied to regenerate and smooth the mesh grids of the simulated body.

The numerical method used in this study is consistent with our previous method [27] on the self-propulsion of the BCF mode. Additional details regarding the setup and method verification can be found in the relevant literature [25]. This numerical method accurately predicts the fluid force and flow field structure, and is widely used in the self-propulsion of virtual fish.

**Figure 5:**
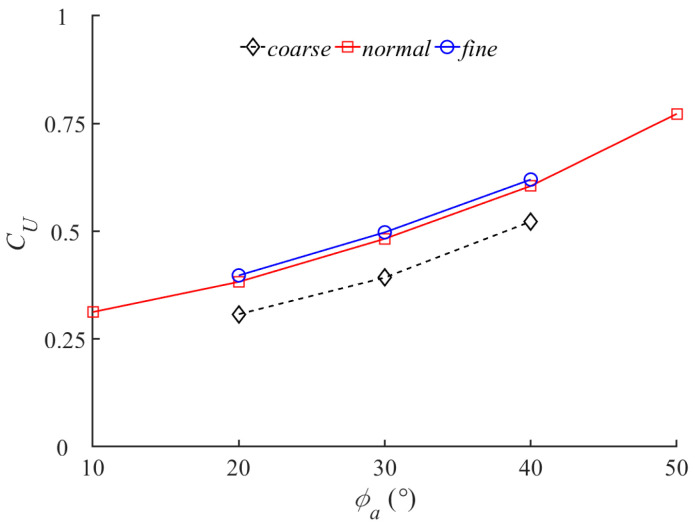
Grid sensitivity test for this numerical method.

This work applies the grid sensitivity test to determine the appropriate grid size. Three sizes of grids with uniform side lengths of 0.016 *L*, 0.008 *L* and 0.004 *L* are applied in the numerical environment, where the corresponding grid numbers are 1.5 (coarse), 5.7 (nominal) and 13.2 (fine) million, respectively. In terms of numerical environment, the domain size and the boundary conditions are unified. In the subsequent quantitative grid sensitivity test, we controlled the flapping angle of the rigid pectoral fin at 20° and the stroking angle of the rigid pectoral fin within the range of 20° to 40°. Figure 5 shows the variation in stable-state propulsion velocity coefficient *C_U_* with the maximum azimuth angle *ϕ_a_*_max_ for three different grid cells. It can be seen that under the same parameter conditions, there is a relatively large error in the swimming law simulated by the coarse grid, while the nominal and fine grids can better deal with the hydrodynamic problems of butterfly swimming. Therefore, a refined uniform grid discretization work area with a size of 0.008 *L* is a suitable choice, which has been reported in the motion analysis of some bionic fish.

In addition, a proper time step is conducive to efficient numerical computation, without distortion of grid node displacement. To control the accuracy of the results and computational cost, we use standard grids to study the time step sensitivity of three conditions (0.01 *T*, 0.005 *T*, 0.0025 *T*). We fix the maximum pitch angle of the rigid pectoral fin at 20°, and set the maximum azimuth angle *ϕ_a_*_max_ within the range of 20° to 40° to control the size of the ellipse trajectory. Table 1 shows the change in stable-state propulsion velocity coefficient *C_U_* with *ϕ_a_*_max_ for three different time steps, where *dt* = 0.005 *T* is proved to be appropriate.

### 2.5. Calculation of Performance Parameters

According to the previously prescribed equations of motion, the self-propelled dolphin relies on the butterfly mode with rigid pectoral fins to drive while keeping the main body fixed. In this study, we focus on straight line motion of the dolphin and apply several calculation parameters to quantitatively regulate self-propelled performance. The components of the instantaneous fluid force in the absolute coordinate system along the advancing direction can be solved by the compressive and shear stresses acting on the virtual dolphin.

(12) $$F_{x}(t)=\int_{S}-pe_{1}dS+\int_{S}\tau_{1j}e_{j}dS$$

where ***e****_j_* is the component of the normal vector on the dolphin body surface d*S*, and *τ_ij_* is the viscous stress tensor in the absolute coordinate system. During the cyclical swimming process of the dolphin, both the differential pressure resistance and friction resistance change periodically. It is judged that the change in the sign of *F_x_*(*t*) during the periodic swimming process will reflect its contribution to the thrust *F_T_*(*t*) and resistance *F_R_*(*t*)

(13) $$F_{T}(t)=\frac{1}{2}\left(\int_{S}-pe_{1}dS+\left|\int_{S}pe_{1}dS\right|\right)+\frac{1}{2}\left(\int_{S}\tau_{1}{}_{j}e_{j}dS+\left|\int_{S}\tau_{1}{}_{j}e_{j}dS\right|\right)$$

(14) $$F_{R}(t)=\frac{1}{2}\left(\int_{S}-pe_{1}dS-\left|\int_{S}pe_{1}dS\right|\right)+\frac{1}{2}\left(\int_{S}\tau_{1}{}_{j}e_{j}dS-\left|\int_{S}\tau_{1}{}_{j}e_{j}dS\right|\right)$$

After defining the thrust and resistance with above decomposition, we can reconstruct the equation of instantaneous net force *F_x_*(*t*) = *F_T_*(*t*) +*F_R_*(*t*). In addition, the power consumed by the dolphin in overcoming fluid interactions in other DoFs through one cycle is considered useless and used as a representation of ${\bar{P}}_{L}$ [12]. To analyze the superiority of butterfly mode driven by rigid pectoral fins, the propulsion process is analyzed according to the Froude propulsion efficiency [28] given as

(15) $$\eta =\frac{{\bar{F}}_{T}U}{{\bar{F}}_{T}U+{\bar{P}}_{L}}$$

where ${\bar{F}}_{T}$ is the average propulsion force of a period in the self-propelled motion, and *U* is the stable propulsion velocity along the advance direction.

To standardize the numerical solution of the virtual dolphin for different types and external dimensions, it is necessary to define the following dimensionless parameters [29]:

(16) $$C_{FT}=\frac{{\bar{F}}_{T}}{0.5\rho U^{2}L^{2}}$$

(17) $$C_{PL}=\frac{{\bar{P}}_{V}}{0.5\rho U^{3}L^{2}}$$

where *C_FT_* is dimensionless propulsion force, and *C_PL_* is dimensionless energy loss. For the dimensionless representation of the performance parameters, we use *C_Fx_*, *u_x_* and *C_U_* to denote the dimensionless longitudinal force, the dimensionless instantaneous forward velocity and dimensionless stable-state propulsion velocity, respectively.

(18) $$C_{Fx}=\frac{F_{x}(t)}{0.5\rho U^{2}L^{2}},\;u_{x}=\frac{\dot{x}(t)\cdot T}{L},\;C_{U}=\frac{U\cdot T}{L}$$

where $\dot{x}(t)$ is the component of instantaneous velocity in the advance direction.

## 3. Results and Discussion

This section focuses on the law of self-propulsion provided by the butterfly flapping mode of the robotic dolphin. As the main propulsion system, A pair of rigid pectoral fins holds three basic attack orientations to perform the flexible butterfly-mode movement. The effective driving force is generated, and the useless span force is balanced.

### 3.1. Time History Variations in Performance Parameters

**Figure 6:**
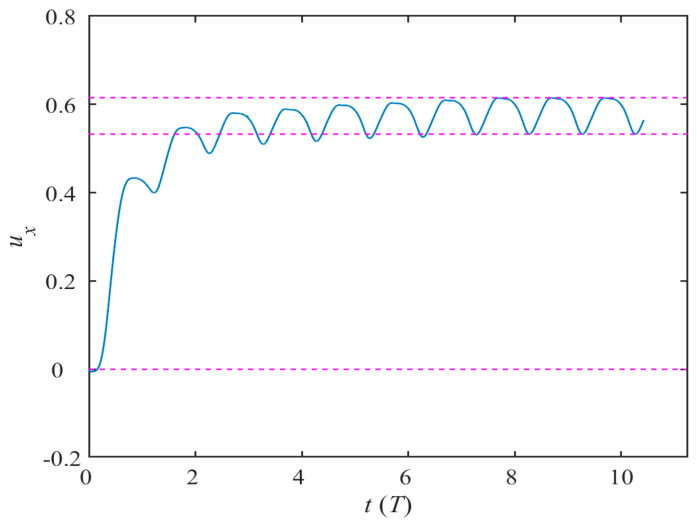
Time history of the transient forward velocity coefficient.

The self-propulsion of the dolphin in the butterfly flapping mode undergoes a dynamic process of gradual convergence. Since the motion form is instantaneously symmetric about the *x_b_y_b_* plane, the motion law in the *oz_b_* axis direction can be ignored. We assume that the stationary dolphin is operated directly from State A in Figure 4, and specify the kinematic performance parameters by defining the water striking frequency *f* = 5 Hz, the maximum pitch angle *ϕ_p_*_max_ = 20° and the maximum azimuth angle *ϕ_a_*_max_ = 40°. The time history of the instantaneous forward velocity coefficient *u_x_* shown in Figure 6 reveals that the bionic dolphin starts from a standstill, gradually accelerates after passing through the preparation phase and finally reaches a steady speed state. During the stable period of time, the instantaneous forward velocity coefficient *u_x_* fluctuates between 0.53 and 0.62, which is different from the sine asymptotic curve exhibited by the BCF self-propelled mode [12]. The curve law converged by the butterfly flapping mode is characterized by sharp narrow valleys and smooth peaks. The specific reasons for the formation will be analyzed in conjunction with hydrodynamics in the following sections.

**Figure 7:**
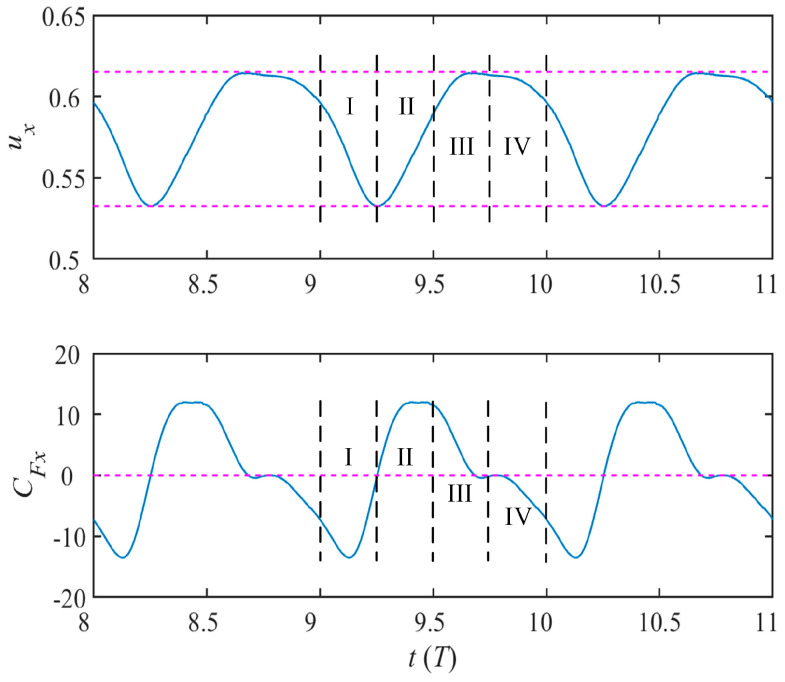
The time history of velocity and force coefficient along the forward direction (The pink dashed lines serves as auxiliary lines, representing the range of numerical fluctuations or reference lines).

After reaching the steady state, the instantaneous forward velocity coefficient *u_x_* and propulsive force coefficient *C_Fx_* along the swimming direction are periodically changed in the form of non-sine waves, as shown in Figure 7. In a single period *T*, the curves of *u_x_* and *C_Fx_* are divided into four regions I, II, III and IV, corresponding to the four morphological changes in the Figure 4. The function of force in the whole process changes from resistance to propulsion, and finally to resistance. The effective propulsion part corresponds to Phases II and III. At this time, pectoral fins moving to the foremost hit the water back in a large area, causing the dolphin to gradually increase *C_Fx_* and produce the maximum propulsion force at (*n* + 3/4)*T*. Phases I and IV correspond to the return of the dolphin’s pectoral fins. Although the water hitting area is small at this time, the ineffective action still affect the propulsion progress and the dolphin obtain the maximum resistance at (*n* + 0)*T*. In the process of the instantaneous force variation, dolphin is in the acceleration phase (II and III) when the force coefficient *C_Fx_* is positive, and the dolphin is in the deceleration phase (I and IV) when *C_Fx_* is negative. Therefore, at the end of the Phase III, the dolphin obtains the maximum propulsion velocity in a single cycle.

### 3.2. Transient Variation in the Flow Field

The transient changes in the flow field surrounding the dolphin help reveal the propulsion mechanism of the butterfly flapping mode. In this section, we capture the main flow structure to display the transient information of motion based on the working condition of the previous section.

**Figure 8:**
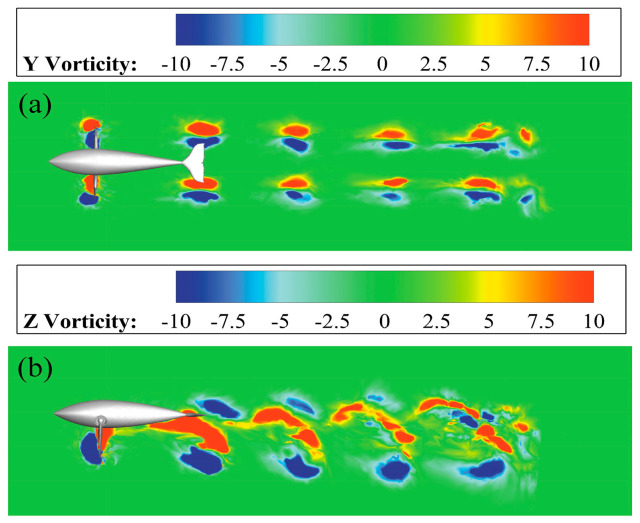
The vorticity contours of butterfly stroke mode. (**a**) The top view; (**b**) The side view.

Figure 8 shows the instantaneous vorticity fields of the two sections (a) and (b) in the stable cruise state, corresponding to the top view of the dolphin and the side view of the right pectoral fin, respectively. Once the robotic dolphin starts to move forward, each cycle of the butterfly movement can produce two pairs of symmetrical and ordered vortex structures downstream of the double pectoral fins (Figure 8a). Figure 8b demonstrates from the *x_b_y_b_* slices that the wake vortex structure is presented with the characteristic double-row vortex law of the butterfly swimming pattern. Two co-directional vortices are located on the center line of the vortex street, and co-directional vortices with the same physical signs are easily merged, while the two opposite vortices are located on both sides. The overall arrangement of vortices is still in the form of a reverse Karman vortex street. The bionic dolphin makes full use of the backward jet in the wake area at the rear edge of the pectoral fins to absorb energy and produce a strong propulsion effect.

**Figure 9:**
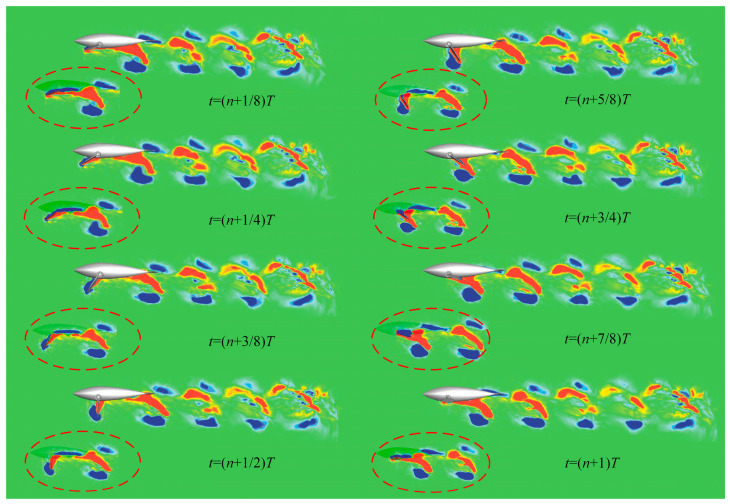
The vorticity contours of butterfly stroke mode within one circle (side view).

Figure 9 shows the vorticity profile of the left pectoral fin within one motion cycle in the *x_b_y_b_* slice, and the details of the vortex shedding blocked by the dolphin’s body are shown in the red dotted frame. The overall pattern of the vortex street changes from the four neat and regular vortex structures in the upstream to the chaotic and scattered vortex distribution in the downstream. When *t* = (*n +* 1/8)*T*, the inner and outer sides of the elliptical trajectory show flow vortices with different rotation directions, which is also called antiphase. When the left pectoral fin moves to the upstream limit of the stroke angle (*t* = (*n +* 1/4)*T*), the red vortex on the inner side falls off firstly. At the next moment, the blue vortex on the outer side also falls off, and the entire shedding path follows the elliptical trajectory of the bionic pectoral fin. From (*n +* 3/8)*T* to (*n +* 5/8)*T*, the vortices attached to both sides of pectoral fins form and grow again. The vortices fall back from the two vortex streets with opposite directions alternately in the downstream stages of resuming the water stroke (*t* = (*n +* 3/4)*T* and *t* = (*n +* 7/8)*T*), where the outside falls off faster than the inside. Because the newly detached inner vortex street is quickly covered by the attached vortex of the flapping pectoral fin, the two same-directional vortices in the middle street merge together in the newly formed cycle of the vortex street, which split laterally in the second cycle. The entire tail space is arranged with staggered double-row wakes. The formation of the four vortices is related to the rapid back and forth motion of the dolphin. They have a phase difference with each other, and they appear in different stages of motion, forming a “vortex dislocation” [30].

### 3.3. Transient Pressure Evolution Process

The butterfly motion based on the elliptical trajectory discussed in this paper undergoes four definite positions. Referring to Figure 3, we determine that Phases I and IV correspond to the return journey of the pectoral fins, which do not contribute significantly to propulsion. Conversely, pectoral fins in Phases II and III experience a substantial and effective push from the water, for time *t* = (*n +* 1/4)*T* to *t* = (*n +* 3/4)*T*. From Figure 7, it can be seen that during this period, the curve of the instantaneous forward velocity coefficient *u_x_* shows a sharp upward trend, while the positive force coefficient *C_Fx_* also reaches its peak value.

**Figure 10:**
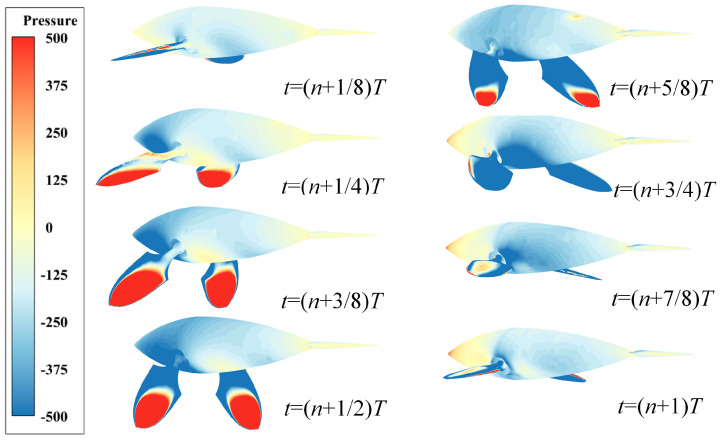
Pressure distribution contours of dolphin surface based on butterfly motion.

To unveil the propulsion mechanism of the butterfly-mode motion based on the elliptical trajectory, Figure 10 portrays the pressure distribution and evolution after the bionic dolphin attains a stable state. By extracting the dynamic pressure distribution on the surface of pectoral fins during a cycle, we note that at time (*n +* 1/4)*T*, the dolphin reaches its anterior limit position (State B in Figure 4). A red high-pressure field forms at the tip of the pectoral fins hitting the water surface, extending longitudinally inward along the fin surface. By time *t* = (*n +* 3/8)*T*, the high-pressure area on the fin’s surface reaches its maximum coverage, corresponding to the peak *C_Fx_* in Figure 7. From (*n +* 1/2)*T* to (*n +* 3/4)*T,* the pectoral fins, having passed through their posterior limit position (State C in Figure 4), gradually release the “high-pressure tide”. This tide eventually recedes and dissipates as the dolphin moves towards its return journey (State D in Figure 4), signifying the formation of a red high-pressure core at the leading edge of pectoral fins. Moreover, due to inertial effects, a high-pressure core forms at the head to counteract fluid reaction forces, while the dolphin’s body and fin surface are covered by a blue low-pressure field. Starting from (*n +* 3/4)*T* to (*n +* 1)*T*, as the pectoral fins approach their upper limit position (State A in Figure 4), a central red-yellow high-pressure region appears on the upper fin surface. Though the strength of the pressure field is not as strong as the hitting point at the leading edge of the fin surface, the combined action of high-pressure regions on the fin surface, leading edge and head determines the bionic dolphin’s force state during this phase.

### 3.4. Effect of Water Striking Frequency on Butterfly Flapping Mode

In this study, the two important parameters that affect the performance of self-propulsion are water striking frequency *f* and butterfly offset angle *ϕ*. The results reveal that they are effective in improving swimming performance and increasing the share of energy consumption during autonomous propulsion.

**Figure 11:**
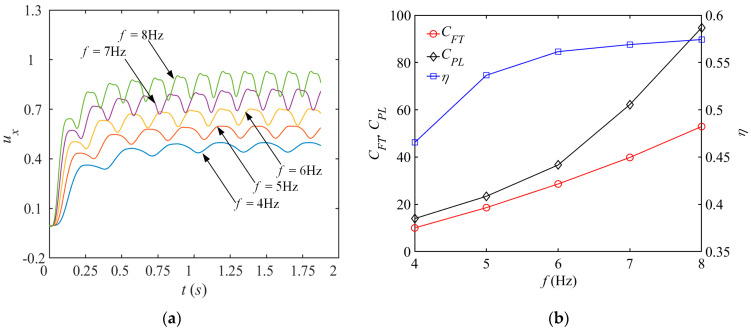
Plots of propulsion performance parameters for different water striking frequencies. (**a**) Relation between *u_x_* and *f*; (**b**) Relation between *C_FD_, C_PL_, η_D_* and *f*.

Figure 11a shows the time history of the instantaneous forward velocity coefficient *u_x_* of the bionic dolphin with different water striking frequencies when other parameters are fixed. It is found that the higher the water striking frequency *f*, the steeper the slope of the *u_x_* curve in the initial stage, indicating a higher acceleration during the transient process. As a result, less time is needed for the dolphin to reach steady state for larger *f*. Moreover, a larger steady-state propulsion velocity coefficient *_U__x_* is found when striking frequency is high. However, with the higher the water striking frequency *f*, increased amplitude of oscillations are observed for *u_x_* in a single cycle. This leads to the less stable motion of the dolphin, indirectly indicating larger energy losses as *f* becomes higher.

To further reveal the mapping relationship between water striking frequency *f* and energetics parameters, we depict the curve law of the propulsion force coefficient *C_FT_*, the average power consumption coefficient *C_PL_* and the propulsion efficiency *η* as function of *f* in Figure 11b. As *f* rises, the propulsion force coefficient *C_FT_* slowly increases, and the average power consumption coefficient *C_PL_* increases with a steeper growth trend after gradually rising. The propulsion coefficient *η* first increases rapidly until the inflection point and then slowly converges. The above analysis with a fixed ellipse trajectory shows that the growth of the propulsion force and efficiency is at the cost of higher energy loss, and increasing the frequency is indeed conducive to the realization of the butterfly swing effect.

### 3.5. Effect of Offset Angle on Butterfly Flapping Mode

During the flapping motion, the pectoral fins of dolphin take the center of the wing root as the base point, and the trajectory of one pectoral fin in one cycle is approximately displayed as an ellipse. In order to facilitate the description of the butterfly mode, we define that the amplitude of the azimuth angle *ϕ_a_*_max_ corresponds to the major axis of the ellipse *a*, the amplitude of the pitch angle *ϕ_p_*_max_ corresponds to the minor axis of the ellipse *b*, and the ratio of the major axis to the minor axis is *r*. To further study the effect of water impact on improving the self-propulsion performance of the virtual dolphin, we assume that the rolling angle is fixed and execute a quantitative ductility test based on *ϕ_p_*_max_ and *ϕ_a_*_max_.

**Figure 12:**
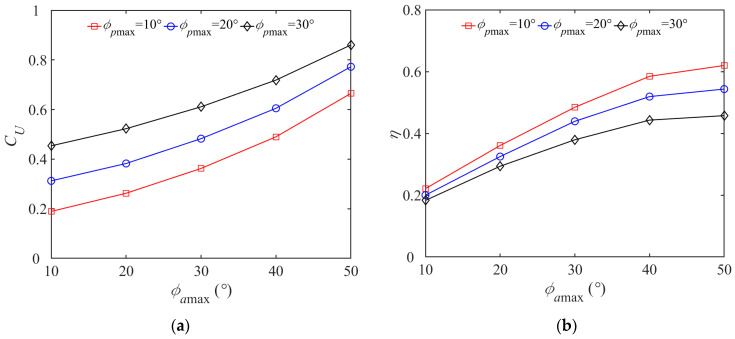
Variations in propulsion performance parameters as functions of offset angle. (**a**) Relation between *ϕ_p_*_max*,*_ *ϕ_a_*_max_ and *C_U_*; (**b**) Relation between *ϕ_p_*_max*,*_ *ϕ_a_*_max_ and *η*.

The combination of various amplitudes of pectoral fin angles leads to elliptical trajectories of different sizes. When *r* is greater than 1, the motion trajectory is a standard elliptical trajectory, and when *r* is less than 1, the trajectory is a vertically inverted ellipse squashed along the *ox_b_* axis. In particular, when *r* is equal to 1, the trajectory is a standard circle. Figure 12a shows the variation in steady-state propulsion velocity *C_U_* with respect to the maximum pitch angle *ϕ_p_*_max_ for different amplitudes of azimuth angle *ϕ_a_*_max_. Specifically, as *ϕ_a_*_max_ increases, the steady-state propulsion velocity *C_U_* increases nonlinearly with an increasing rate of change. It can be seen that the monotonous increase in *ϕ_p_*_max_ and *ϕ_a_*_max_ helps improve the steady-state propulsion velocity *C_U_* of the dolphin. When the values of both parameters are small, *ϕ_p_*_max_ takes a larger share in the enhancement of the propulsion effect. When both values are larger, the change in *ϕ_a_*_max_ causes more change in *C_U_*.

The variation law of propulsion efficiency calculated according to the Froude theory is shown in Figure 12b. For a given maximum pitch angle *ϕ_p_*_max_, *η* gradually increases with the growth of *ϕ_a_*_max_, where its rate of change decreases. A reasonable explanation is that a larger azimuth angle is accompanied by more self-rotation around its own axis. This can generate a longer stroking distance to produce larger angle of attack and more effective water hitting area, which is conductive to increasing *η*. Careful observation reveals that for a given larger azimuth angle *ϕ_a_*_max_, as *ϕ_p_*_max_ rises, *η* decreases drastically. This conclusion is also applicable when *ϕ_a_*_max_ is small, but the effect on decreasing *η* is not significant. The perimeter of the elliptical trajectory formed by *ϕ_p_*_max_ and *ϕ_a_*_max_ in a period is directly proportional to the parameter value of the forward speed in the steady state. In terms of propulsion efficiency, *ϕ_a_*_max_ plays a dominant role in the propulsion, while *ϕ_p_*_max_ acts as an indispensable but adverse role. Therefore, we can choose to increase *ϕ_a_*_max_ and reduce *ϕ_p_*_max_ to enhance the propulsion efficiency.

### 3.6. Three-Dimensional Flow Structure of Butterfly Stroke Mode

**Figure 13:**
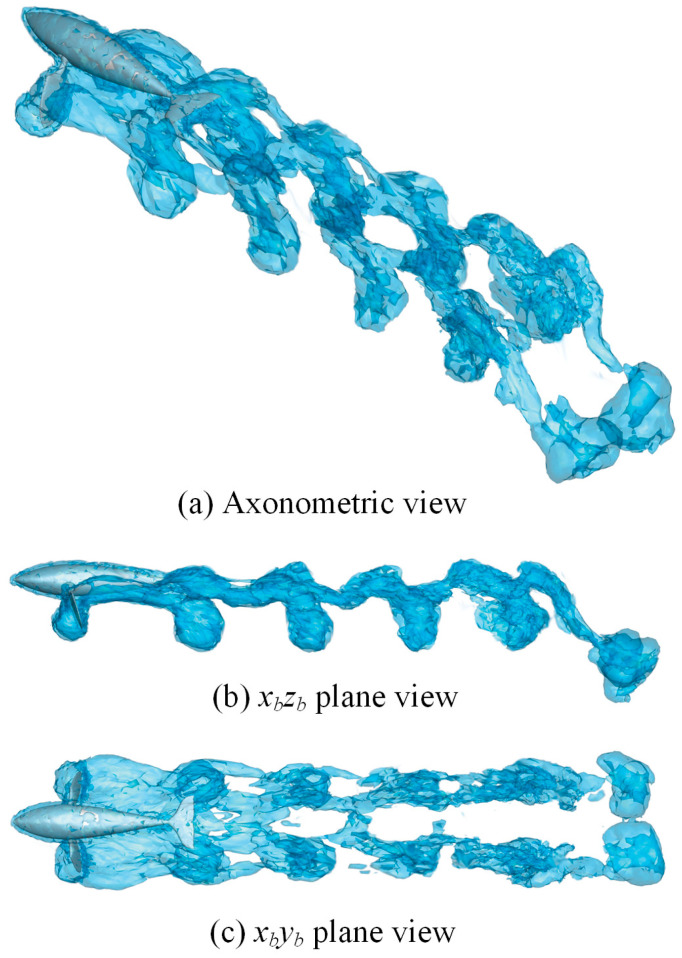
The vorticity iso-surfaces when *f* = 5Hz, *ϕ_p_*_max_ = 20°, *ϕ_a_*_max_ = 40°.

Section 3.2 presents the 2D wake structures, revealing the propulsion performance of the butterfly motion from the details of the vorticity profile. In this section, we use the *q*-criterion to extract the 3D vortex iso-surface of the elliptical trajectory. Based on the kinematic law of Figure 4, we depict the vorticity iso-surface from three perspectives in Figure 13. The entire vortex layout presents a series of interlocking vortex loops. The vortex structure in a single cycle corresponds to a single sickle-like shape. It is a novel type of vortex that has not been reported, which is different from the elongated “hairpin” vortex ring generated by the caudal fin swings [12].

**Figure 14:**
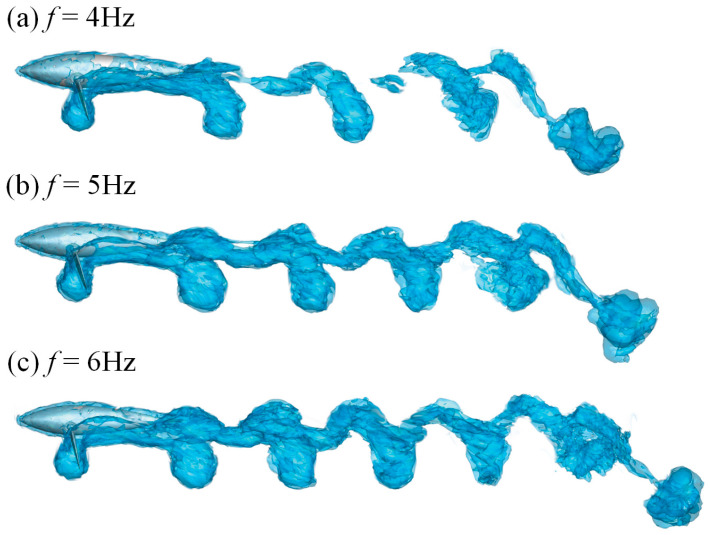
The vorticity iso-surfaces based on different swing frequencies of the pectoral fins.

In Figure 14, we compare the vortex law of the butterfly flapping mode on *x_b_y_b_* plane with different swing frequencies of the pectoral fins. The aligned vortices arranged from upstream to downstream have a similar sickle-like shape, which form a half-ellipse shape as the rigid pectoral fins move. As the motion frequency increases from 4Hz to 6Hz, the intensity of the vortex gradually increases and the axial spacing of a single wake gradually decreases. When the vortices detach more quickly from the lower edge of pectoral fins, the pressure difference in the surrounding flow field becomes more significant, which produces greater propulsion effect on the entire dolphin. Additionally, the energy loss at the wake declines, and the effective propulsion power rises, indirectly explaining the influence of frequency variation on the propulsion efficiency.

**Figure 15:**
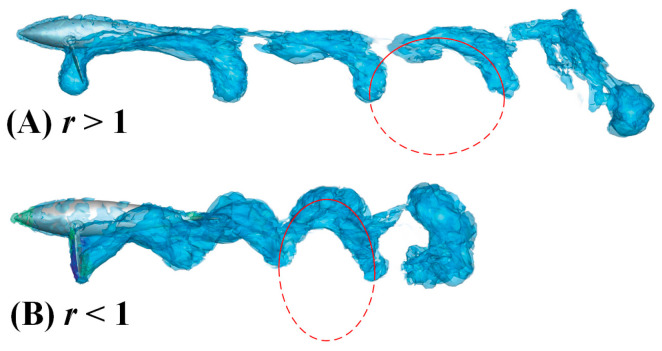
The vorticity iso-surfaces based on different *r*.

To reveal the effect of the ellipse trajectory produced by the offset angle on the wake structure, we keep the water striking frequency *f* and the minor axis *b* unchanged and adjust the size of the major axis *a* in the ellipse trajectory to control the ratio *r*. In Figure 15, we select two working conditions *r* = 5/3 and *r* = 1/3 to draw iso-surfaces. Careful observation reveals that the vortex structures with different *r* values follow the vortex street characteristics of elliptical trajectories. That is to say, when *r* is greater than 1, the vortex structure is a standard ellipse, otherwise it is an inverted one. After comparing the two working conditions, we find that the width of the vortex in the span direction is approximately the same at the initial stage of shedding due to the constant value of the minor axis. In the later stage of shedding, due to the high propulsion speed, the scroll structure in Figure 15A has more components along the forward direction, and the vortex rings are separated from each other. In Figure 15B, the vortex ring spacing is not as large as that in Figure 15A, which causes energy loss along the lateral direction. Therefore, the propulsion efficiency of working condition (B) is not as good as (A).

## 4. Conclusions

The hydrodynamic effect of the butterfly-mode flapping propulsion achieved by a pair of rigid pectoral fins has been systematically investigated. The details of the study mainly include the time history analysis of important parameters under one classic working condition, the influence of quantitative variable parameters on the propulsion effect and the law provided by the instantaneous vorticity structure. The main findings can be summarized as:

(1) The advancing function of the butterfly mode is a transient process that gradually converges. The kinematics of pectoral fins can be described by an elliptical trajectory. In Phases II and III, the effective working area of the large backward water push is beneficial to quickly increase the propulsion speed, and the return of the pectoral fin is bound to bring resistance and energy loss.
(2) Water striking frequency *f* and offset angle *ϕ* are two control parameters used to quantitatively describe the law of butterfly stroke. On one hand, increasing the frequency is indeed conducive to the realization of the butterfly motion effect. On the other hand, the monotonous increase in *ϕ_p_*_max_ and *ϕ_a_*_max_ helps to improve the steady-state propulsion velocity coefficient *C_U_* of the dolphin. In terms of the propulsion efficiency, *ϕ_a_*_max_ plays a dominant role, while *ϕ_p_*_max_ acts as an indispensable but adverse role. So within the range of parameters studied in this paper, the working condition (*ϕ_p_*_max_ = 10°,*ϕ_a_*_max_ = 50°) is the best choice for effective propulsion. In addition, based on this working condition, if we increase the *ϕ_p_*_max_ to 30°, the dolphin could maintain a faster propulsion speed but with slightly lower efficiency.
(3) The butterfly-mode propulsion can produce double rows of vortex streets downstream of the double pectoral fins. In addition, a “vortex dislocation” is formed between individual vortices, where the distance between each periodic vortex is determined by the water striking frequency *f* and offset angle *ϕ*. The entire shedding path follows the elliptical trajectory of the bionic pectoral fin, so the size of the sickle-shaped vortex depends on the size of the major and minor axis of the ellipse.

In this study, a virtual dolphin swimmer is successfully constructed to realize the butterfly flapping mode, and the hydrodynamic performance under self-propulsion is investigated. This motion mode, driven by pectoral fins, extends some previously unreported flow field information. In addition, the simulation of the novel propulsion mechanism proves that not only the caudal fin, but also rigid pectoral fins can act as effective tools for the dolphin’s maneuver under water. Because dolphins have complex fin–fin movements, it is necessary for us to evaluate the interaction of composite fins and explore the effect of optimized fin flexibility on locomotion performance in follow-up studies.

## Figures and Tables

**Table 1:** Variations in the steady-state propulsion velocity coefficient *C_U_* with the maximum azimuth angle *ϕ_a_*_max_ for different time steps.

*ϕ_a_*_max_ (°)	10	20	30	40	50
*dt* = 0.01 *T*	——	0.33	0.42	0.53	——
*dt* = 0.005 *T*	0.31	0.38	0.48	0.60	0.77
*dt* = 0.0025 *T*	——	0.39	0.50	0.62	——

## Data Availability

All data are available in the main text.
